# Blood flow characteristics of diabetic patients with complications detected by optical measurement

**DOI:** 10.1186/s12938-018-0457-9

**Published:** 2018-02-21

**Authors:** Yuri An, Yujung Kang, Jungsul Lee, Chulwoo Ahn, Kihwan Kwon, Chulhee Choi

**Affiliations:** 1 Department of Bio and Brain Engineering, KAIST, and Cellex Life Sciences, Inc., Daejeon, Republic of Korea; 2 R&D Center, Vieworks Co., Anyang-si, Gyeonggi-do Republic of Korea; 3 Cellex Life Sciences, Inc, Daejeon, Republic of Korea; 4 Department of Internal Medicine, Yonsei University College of Medicine, Seoul, Republic of Korea; 5 Department of Internal Medicine, College of Medicine, Ewha Womans University, Seoul, Republic of Korea

**Keywords:** Diabetes mellitus, Diabetic complications, Blood flow characteristic, Medical imaging, Indocyanine green

## Abstract

**Background:**

Diabetes mellitus (DM) is one of the most common diseases worldwide. Uncontrolled and prolonged hyperglycemia can cause diabetic complications, which reduce the quality of life of patients. Diabetic complications are common in DM patients. Because it is impossible to completely recover from diabetic complications, it is important for early detection. In this study, we suggest a novel method of determining blood flow characteristics based on fluorescence image analysis with indocyanine green and report that diabetic complications have unique blood flow characteristics.

**Methods:**

We analyzed time-series fluorescence images obtained from controls, DM patients, and DM patients with complications. The images were segmented into the digits and the dorsum of the feet and hands, and each part has been considered as arterial and capillary flow. We compared the blood flow parameters in each region among the three groups.

**Results:**

The DM patients with complications showed similar blood flow parameters to the controls, except the area under the curve and the maximum intensity, which indicate the blood flow volume. These parameters were significantly decreased in DM patients with complications. Although some blood flow parameters in the feet of DM patients with complications were close to normal blood flow, the vascular response of the macrovessels and microvessels to stimulation of the hands was significantly reduced, which indicates less reactivity in DM patients with complications.

**Conclusions:**

Our results suggest that DM patients, and DM patients with complications, have unique peripheral blood flow characteristics.

## Background

In diabetes mellitus (DM) patients, limb perfusion monitoring is critical due to its vulnerability to peripheral macro- and micro-vascular complications [1, 2]. Because a high glucose level can induce vascular endothelial cell dysfunction and affect blood viscosity and arterial wall tension [3–5], DM patients are at higher risk for development of vascular complications than non-diabetic persons, and the frequency of limb amputation is 23-fold higher in DM patients than in normal controls [6, 7]. In DM patients, coronary artery disease develops more easily and rapidly [8], which is a life-threatening disease [5, 9, 10]. Moreover, diabetic microvascular complications affect coronary arterial disease, which is the most important long-term complication and the most common cause of death in diabetes [11]. Therefore, monitoring and understanding the blood flow characteristics of DM patients is important.

Indocyanine green (ICG) is a fluorescent dye that has been used in clinics for decades and it is activated by near-infrared (NIR) light, which can penetrate to a depth of several centimeters. Using ICG imaging, blood perfusion in deep tissue can be monitored. When introduced into the systemic circulation, ICG binds to plasma proteins such as albumin [12] and circulates through the vasculature. The vascular properties of tissue can be extracted by dynamic analysis of the fluorescence kinetics of ICG. ICG fluorescence imaging has been used to detect various diseases, such as synovitis [13, 14], rheumatoid arthritis [15–17], peripheral vascular disease (PVD) [18, 19], and diabetes [7, 20, 21]. Compared to other blood flow characterization systems, such as laser Doppler imaging (LDI), ICG fluorescence imaging has several advantages. LDI is the technique basically measuring velocity of a red blood cell (RBC) [22], not the blood perfusion. However, with ICG imaging, blood perfusion can be monitored [23]. Moreover, blood permeability can be measured by ICG imaging, [24] and ICG imaging has greater sensitivity for monitoring tissue perfusion [23]. LDI imaging can show detailed information in a small area and is adequate for tissue perfusion [25, 26], but not for imaging of whole organs due to the long acquisition and processing times [22]. Compared to LDI, ICG imaging had already been used for even whole body imaging [27, 28].

Due to such a high demand for understanding the blood flow characteristics in diabetic patients, in this study, we characterized the blood flow of DM patients with and without complications by fluorescence imaging with ICG.

## Methods

### Volunteers

All protocol, data, and consent forms for human subjects in this research were approved by the Institutional Review Board of Gangnam Severance Hospital (Seoul, Korea). All experimental procedures were conducted according to the principles of the Declaration of Helsinki. Written informed consent was obtained from each subject. The control group (C) comprised volunteers who had no history of DM, inflammatory disease history, or vascular disease, and had no other vascular risk factors (Table 1). DM only group (D) comprised DM patients with no history of microvascular or macrovascular disease. The DM patients with complications group (M) comprised patients with a diabetic complications, such as retinopathy or neuropathy, which was diagnosed by experienced physicians [29, 30].

**Table 1:** Demography of each group

Groups	Control	DM	M	*p* value
n	14	11	16	–
Age (years, mean ± SD)	55.5 ± 7.2	62.2 ± 6.5	58.6 ± 6.0	n.s.
Median	56	65	59	–
Gender (male/female)	2/12	6/5	9/7	< 0.05
DM duration (years, mean ± SD)	–	8.7 ± 6.0	16.2 ± 9.4	< 0.05
DM complication duration (years, mean ± SD)	–	–	3.7 ± 4.0	–
HTN (n)	1	4	6	–
ABI (mean ± SD)	1.15 ± 0.06	1.18 ± 0.05	1.15 ± 0.06	n.s.
PWV (cm/s, mean ± SD)	1300 ± 96	1674 ± 417***	1560 ± 221***	< 0.0001
HbA1c (mean ± SD)	5.6 ± 0.4	6.6 ± 0.5*	7.6 ± 1.5***	< 0.0001
Smoking (n)	0	1	4	–
Dyslipidemia (n)	1	2	6	–

*HTN* hypertension, *ABI* Ankle-brachial index

Values were subjected to one-way analysis of variance (ANOVA) with a post hoc Bonferroni test; * *p* < 0.05, *** *p* < 0.001, compared with control; Chi square was used for analyzing gender

### ICG imaging

**Fig. 1:**
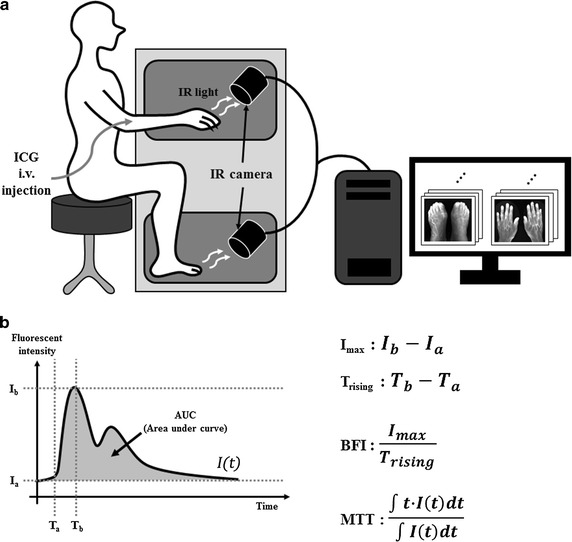
The ICG imaging system. **a** Indocyanine green (ICG) imaging scheme. ICG is bolus-injected intravenously, and near-infrared (NIR) imaging acquisition begins simultaneously. The fluorescence images were analyzed using the respective vascular tracer parameter calculation algorithms. **b** Calculation algorithms for the vascular tracer parameters: I_max_, difference between the maximum and minimum fluorescence intensities; T_rising_, time from onset to I_max_; BFI, the I_max_ to T_rising_ ratio. MTT (mean transit time) is calculated as shown

For ICG time-series imaging, 120 images (768 × 512 pixels) of the dorsum of both feet and hands were taken at 5-s intervals for 10 min simultaneously with an intravenous bolus injection of ICG (0.16 mg/kg). ICG fluorescence images were obtained using a NIR imaging system equipped with a charge-coupled device (CCD) digital camera with an 830-nm bandpass filter and 760-nm light-emitting diode arrays (VISQUE, Vieworks Corp., Anyang, Gyeonggi-do, Korea), as previously reported [18, 20, 31–33]. A schematic diagram of ICG imaging is shown in Fig. 1a.

### Fluorescent image analysis

Regional ICG dynamics obtained from each pixel was analyzed to obtain the I_max_ value, blood flow index (BFI), mean transit time (MTT), and the area under the curve (AUC). I_max_ is the maximum fluorescence intensity after subtraction of the basal intensity, and the AUC is calculated as the area under the curve without baseline intensity area; both are indirect indicators of the blood volume in the region of interest (ROI) [34, 35] (Fig. 1b). The BFI is the ratio of ΔI and ΔT, which indicates the slope of the fluorescence intensity rising curve [36]. MTT is the average time from blood in and out at a specific pixel, and is calculated as shown in Fig. 1b [37]. Every parameter calculation method is shown in Fig. 1b. To analyze regional time-series pharmacokinetics, we used Visual C++ (version 10.0 SP1, Microsoft, Redmond, WA). The analyzed region was divided into toes and dorsum. The toe part represents peripheral arterial input and the dorsum part represents microvascular circulation, as reported previously [20, 32]. To extract the vascular tracer parameters from each region, we collected time-series pixel intensities as single vectors. Each value was collected from each foot and hand separately. The images of volunteers who moved their digits, hands, or feet by > 1 cm during the initial 3 min of imaging were excluded because of the movement artifact.

### Vascular stimulation

All volunteers underwent imaging in a resting state and after vascular stimulation. Vascular stimulation was induced by pressing the brachial artery with an air cuff, which is a commonly used method for endothelial function testing [38]. After 3 min of cuff stimulation, volunteers rested for 1 min and then underwent a second round of ICG imaging.

## Results

### Volunteers’ characteristics

The demographic characteristics of the volunteers are shown in Table 1. The groups were age matched. The number of female subjects was greater than that of male subjects in control, and vice versa in the M group. Pulse wave velocity (PWV), which is blood velocity by arterial pulse propagation, is shown in Table 1. PWV represents arterial stiffness [39, 40], and is strongly correlated with cardiovascular disease [41, 42]. Its measuring method is described in previous studies [43]. The PWV and HbA1c values differed significantly among the three groups (one-way ANOVA, *p* < 0.0001).

### Blood flow characteristics

**Fig. 2:**
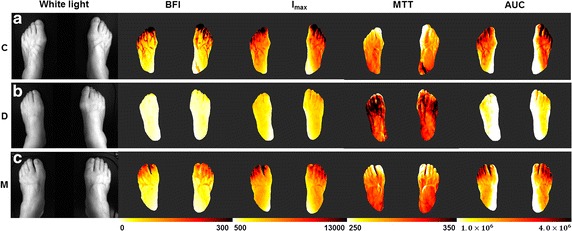
Representative vascular tracer parameter maps. Representative vascular tracer parameter maps of the control (**a**), diabetes mellitus (D) (**b**), and diabetic patients with complications (M) (**c**) groups. The first column shows white light images of each foot

**Fig. 3:**
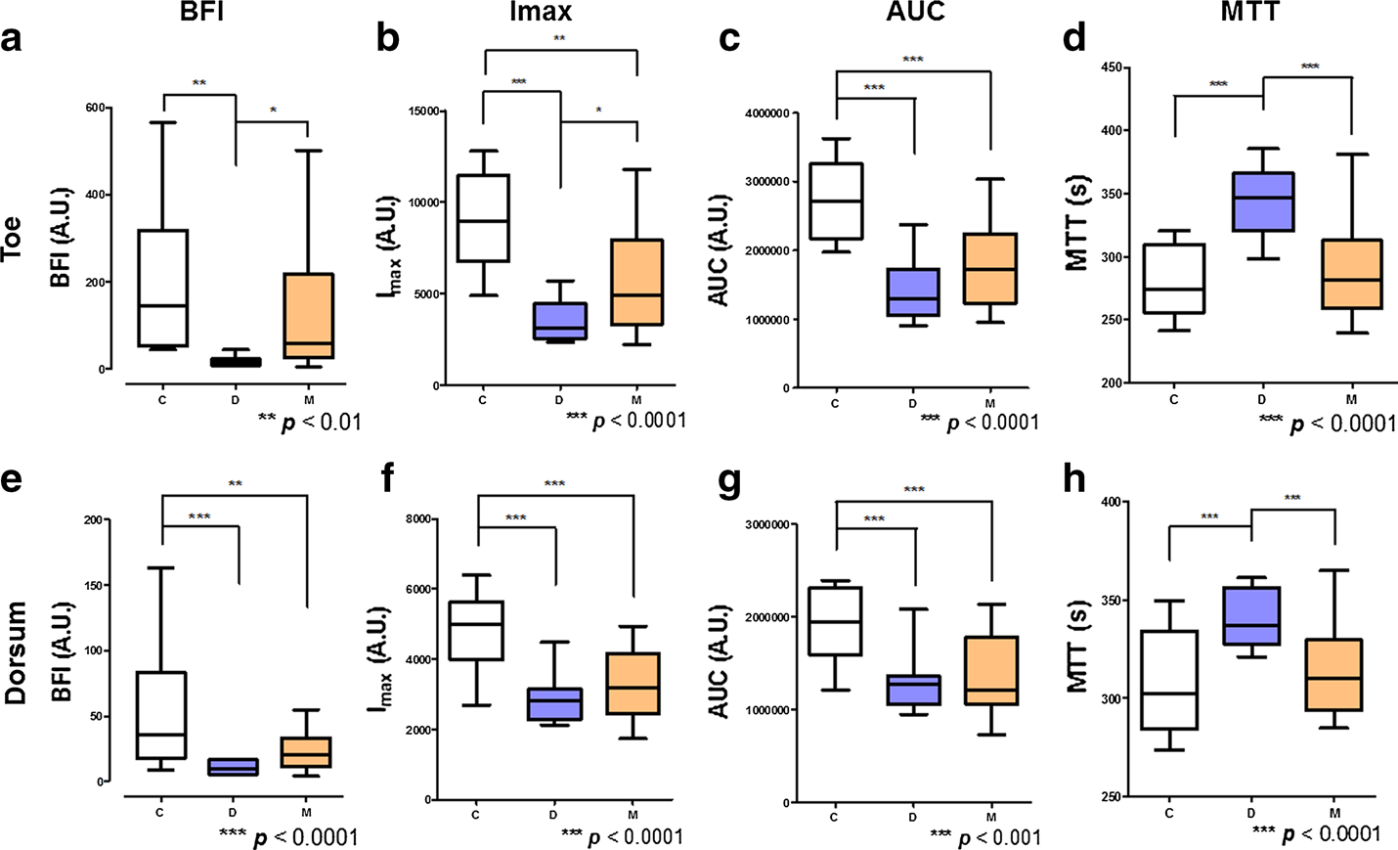
Vascular tracer parameters. Top panels, toes; bottom panels, dorsum. Blood flow index (BFI) (**a**, **e**), I_max_ (**b**, **f**), area under the curve (AUC) (**c**, **g**) and mean transit time (MTT) (**d**, **h**) values. Values were subjected to analysis of variance (ANOVA) with a post hoc Bonferroni test. The values of all parameters in the toes and dorsum in the D group were significantly different from those of the C group. The M and C group showed a similar pattern of BFI in toes and MTT; however, the BFI value in the dorsum part, and the I_max_ and AUC values in both parts, differed significantly between the C and M groups. The D group showed a delayed MTT value (**c**, **g**) (*p < 0.05, **p < 0.01, ***p < 0.001)

The BFI, I_max_, AUC, and MTT values are shown in Table 2, Figs. 2 and 3. The C and M groups showed similar BFI and MTT values compared to the D group. However, the I_max_ and AUC values of both parts, and the BFI value of the dorsum part, differed significantly between the C and M groups. The C and M groups exhibited higher BFI values in the toes compared with the D group, indicating a rapid and considerable increase in blood flow (Fig. 3a). Also, the D group showed a significantly higher MTT value than the C and M groups. Therefore, the mean circulation time in the D group was delayed than that in the C and M groups.

**Table 2:** Blood flow parameters of each group at foot

Groups	C	D	M	*p* value
BFI				
Toe (A.U., mean ± SD)	199.4 ± 167.2	17.1 ± 10.9**	124.0 ± 135.6	< 0.005
Dorsum	52.8 ± 43.4	10.6 ± 5.1***	24.0 ± 15.1**	< 0.0001
I_max_				
Toe	8967.7 ± 2700.0	3519.3 ± 1139.8***	5798.9 ± 2742.9**	< 0.0001
Dorsum	4750.4 ± 1092.9	2907.3 ± 729.9***	3313.4 ± 960.9***	< 0.0001
MTT				
Toe	279.3 ± 26.0	344.6 ± 27.6***	290.4 ± 37.0	< 0.0001
Dorsum	308.1 ± 24.0	341.6 ± 15.1***	312.9 ± 22.7	0.0001
AUC				
Toe	2,736,757 ± 544,287	1,448,562 ± 479,210***	1,774,164 ± 598,795***	< 0.0001
Dorsum	1,917,892 ± 385,771	1,309,075 ± 333,641***	1,362,036 ± 413,776***	0.0002

Values were subjected to one-way ANOVA with a post hoc Bonferroni test; ** *p* < 0.01, *** *p* < 0.001, compared with control

In contrast, the AUC and I_max_, values differed between the C and M groups. This suggests that DM patients with complications have more rapid blood flow but lower absolute blood volume than healthy controls. In the C and M groups at toes, blood flow was faster than that in the D group, and in M group, the blood volume was less than that in the C group. The D group had the lowest blood flow and the lowest absolute blood volume; the dorsum part exhibited a similar pattern to that in the toe part, with the exception of BFI in the dorsum part (Fig. 3e), which suggested rapid blood flow only in the C group.

### Response to stimulation

**Fig. 4:**
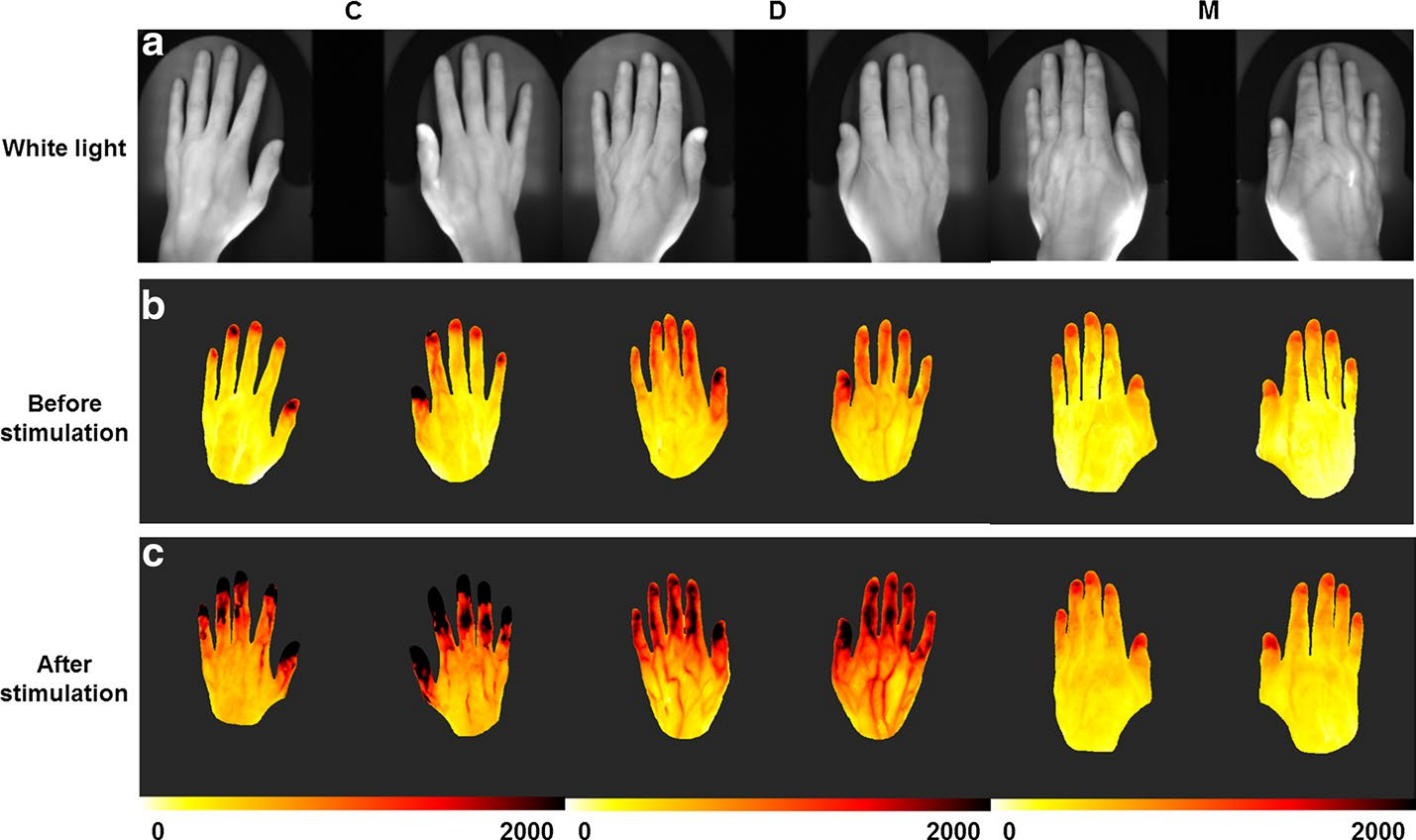
BFI maps before and after cuff stimulation. Representative BFI maps before (**b**) and after (**c**) stimulation of the C, D, and M groups. The first row shows white light images (**a**) of each hand

**Fig. 5:**
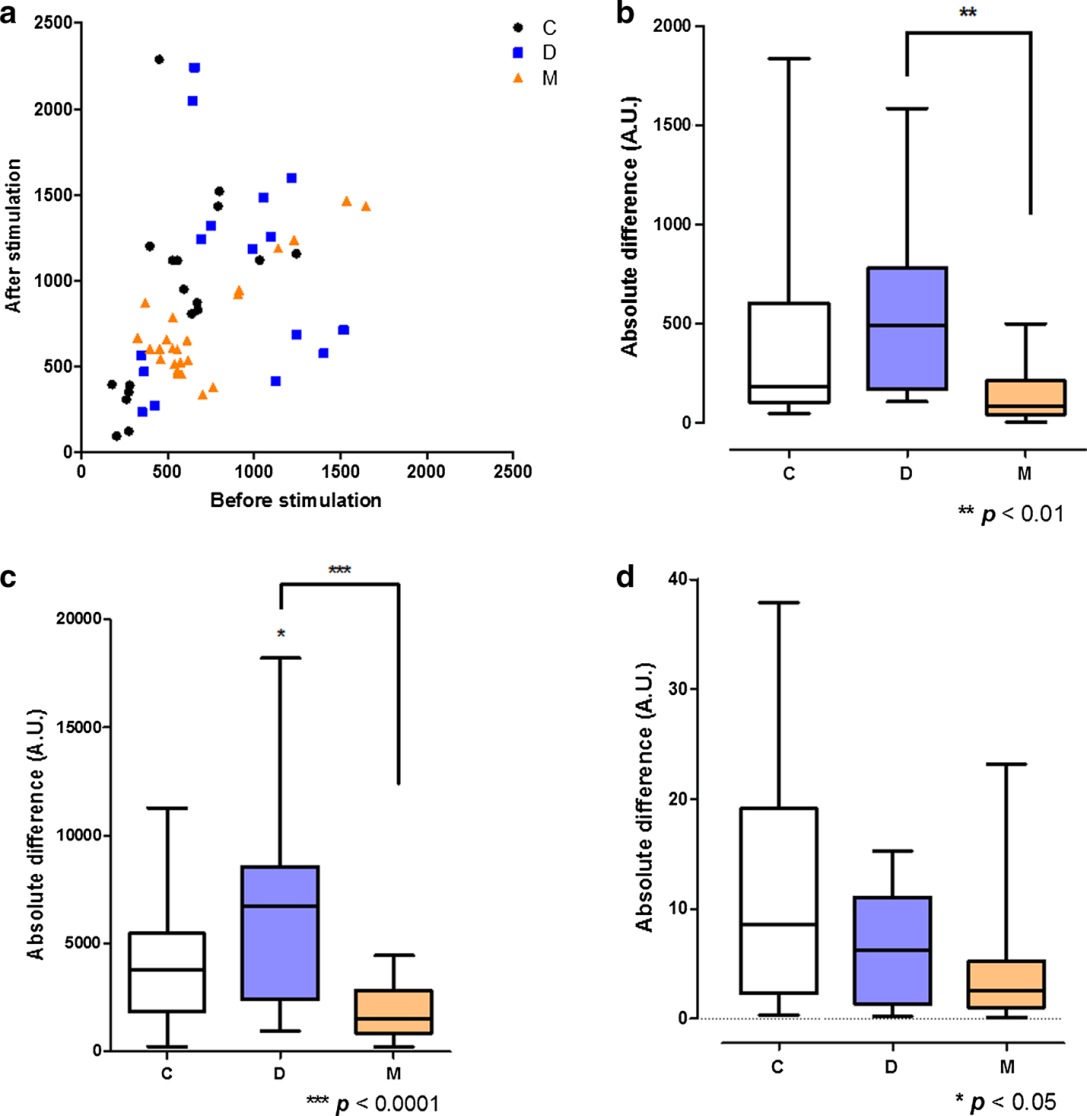
Response to stimulation. **a** X-axis, BFI values in the fingers before cuff stimulation; y-axis, BFI values after cuff stimulation. Absolute difference in BFI values (**b**), I_max_ values (**c**), and T_rising_ values (**d**) in the fingers before and after stimulation. Note that the M group did not show a response to stimulation. Values were subjected to ANOVA, with a post hoc Bonferroni test

The volunteers were subjected to a stimulus, followed by a flow-mediated dilation (FMD) protocol [38], and were measured difference of the blood flow characteristics with ICG fluorescent imaging. The C and D groups, but not the M group, showed a response to the stimulus in the hands (Figs. 4, 5). In the C group, BFI increased after application of the stimulus. A portion of the D group showed an increased BFI value, and the remainder a decreased BFI value. However, in the M group, the BFI value was unaffected by the stimulus, as indicated by the closeness of the BFI values to the *y* = *x* line (Fig. 5a). Figure 5b shows the absolute BFI difference before and after stimulation. The BFI value varied little in the M group before and after application of the stimulus. We investigated which parameter of BFI effected to this phenomenon (Fig. 5c, d). The differences in the I_max_ and T_rising_ values, which are all related with BFI, before and after stimulation were of significantly lesser magnitude in the M group than the other two groups. Therefore, even though the stimulus can induce blood flow increase and vasodilation, in the M group the stimulus did not induce a blood-flow response in the upper extremities. No such phenomenon was detected in the lower extremities (data not shown) as the cuff stimulus was applied to the upper arm, and endothelial function in the brachial artery does not reflect that in the tibial artery [44]. Therefore, lower extremities did not show significant response before and after stimuli.

## Discussion

This study aimed to determine the blood flow characteristics of diabetic patients with complications and to suggest appropriate diagnostic values. Diabetes has a strong association with both micro- and macro-vascular complications [45]. Diabetic retinopathy, neuropathy, and nephropathy has been categorized as diabetic microvascular complications in decades [2, 11, 46]. When diabetic neuropathy or retinopathy is present in diabetic patients, the progress of diabetes is more advanced, so it can be said that microvascular dysfunction becomes worse [11]. In this study, we considered diabetic retinopathy or neuropathy as a marker that shows severity of diabetes, and due to the increased disease severity, we considered that diabetic patients with these complications could have more severe microvascular dysfunctions than the diabetes only patients. With this grouping criterion, we divided patients into three groups, which are control, diabetic patients, and diabetic patients with complications.

We suggested that ICG imaging could monitor these diabetic complications in early stage and it could show the blood flow characteristics of each group. Our result has shown distinct features compared to previous researches with NIR imaging [7, 47]. We initially expected that blood flow would be slowest and blood volume lowest in DM patients with complications, followed by DM patients and controls. However, the result was not matched to our assumption. Blood flow velocity and volume were lowest in the DM only group, which is considered as intermediate-stage of disease prognosis. Through Figs. 2, 3, we could find out the blood flow pattern of each group. The C group showed the fastest blood flow and largest blood volume in the feet, both arterial and capillary flow. This blood flow is retarded and reduced in D group. Blood flow was higher in the M group than the D group, but blood volume was not, especially in the dorsum part, likely due to a reduced vasodilatory capacity. In the dorsum part, which reflects the blood flow of capillaries, BFI value is significantly decreased in the M group compared to the C group, not like in the toe part. The toe/brachial index (TBI) was significantly lower in D group than in the C and M groups, in previous study [48]. Also, in diabetic complications patients, transcutaneous oxygen (TcPO_2_) was significantly different due to patient’s position, likely due to the disturbance of vessel autoregulation by autonomic neuropathy [49]. From these factors, it can be assumed that microvascular vessel control fails due to vascular complications, which reduces vascular resistance, in M group. Therefore, the BFI and MTT values of the M group were similar to those of the control group, but it does not mean that C and M groups have the same blood flow characteristics.

Moreover, it has been reported in several studies that DM leads to vascular dysfunction, even in the absence of microvascular complications [3, 50, 51]. Blood flow is insufficient in early DM patients but is faster in DM patients with microvascular complications [50, 52, 53]; this is in agreement with our finding of slower blood flow and a lower blood volume in the D group. In the presence of diabetic peripheral arterial disease (PAD), the PWV is in the normal range, whether DM without PAD patients showed higher PWV value [54]. This is in agreement with our data (Table 1). Thus, vascular tone is disrupted in DM patients with complications, and so all vascular shunts are open and vascular resistance is reduced [48, 49, 55]. Therefore, in DM patients, peripheral limb blood flow dramatically decreases because of vessel damage due to hyperglycemia. It could be said that even there is no diabetic complications, DM patients seemed to have microvascular insufficiencies. Then, when vascular disturbance has been exacerbated to get microvascular problems, it can be assumed that arteriovenous fistula is occurred, which reduces vascular resistance. Although the vascular resistance is decreased, blood circulation is impaired by damage due to hyperglycemia, and the total blood volume does not recover.

When there is a vascular disorder such as endothelial dysfunction, it is well known that people do not properly response to the vascular stimulations [38, 43]. Because DM patients are vulnerable to vascular disorder and endothelial dysfunction [1, 3, 5, 56], they do not react properly to stimuli. In this study, we found that DM patients with and without complications react to vascular stimuli differently (Figs. 4, 5). The DM patients with complications showed a vascular response of lesser magnitude than the controls or the DM patients in upper extremities. Regarding the BFI values before and after stimulation, the largest variance was in the C group and the lowest in the M group (Fig. 5). This was a distinguishing feature of DM patients with complications.

Interestingly, some patients in the DM group showed an increased BFI value after stimulation, but two patients (both hands) showed a decreased BFI value (Fig. 5a). The only common feature of two patients is hypertension. It is well known that DM patients have an impaired macrovascular response [57, 58], but only two of our patients showed an impaired vascular blood flow response. This is likely because ICG imaging reveals not only macrovascular endothelial cell reactions but also the microvascular systemic reaction and endothelial cell reactions. In addition, autonomic dysfunction, which is related to expansion of microvascular vessels, in DM patients may also explain their post-stimulation blood flow characteristics. The FMD and nitroglycerin-mediated dilation tests are typically used to assess vascular endothelial cell reactions [43]; these reflect the reactivity of mainly macrovascular endothelial cells [59]. However, endothelial cell dysfunction is a systemic process, in which both macrovascular and microvascular reactivity should be considered. Moreover, the FMD test has several limitations that FMD methodology needs to be standardized and probe position can dramatically affect the result [43]. Therefore, our ICG imaging method can be used for macro- and micro-vascular endothelial cell function testing, and for assessing autonomic dysfunction. Although our findings should be verified in further studies, our method shows promise for evaluating the responses of vascular endothelial cells.

We have found several blood flow characteristics of patients with diabetes and diabetic complications, but this study had several limitations. Due to the small population and the nature of DM, risk factors for vascular disease—such as smoking, dyslipidemia, and hypertension—could not be adjusted for. In addition, all of the DM patients were taking diabetes medications, and some of the controls were taking medications for non-vascular diseases.

## Conclusions

We report herein a significantly different blood flow pattern in DM patients with and without complications by ICG fluorescent imaging. DM only patients have vascular disruption despite the absence of symptoms of vascular disease; this was reflected in ICG fluorescent images. In DM patients with complications, ICG time-series dynamics showed normal-mimicking patterns which are similar with that in the controls, but the blood volume was lower than that in the control. The more rapid blood flow in DM patients with than without complications may be due to autosympathectomy, which may lead to vascular shunt opening and reduction in vascular resistance.

We report here a novel vascular endothelial cell function test based on ICG fluorescent imaging. Although the underlying mechanism is unclear and further clinical study is necessary, our method enables measurement of not only macrovascular reactions but also microvascular and systemic reactions.

In this study, we analyzed the ICG images with dividing the image as segments of arterial blood flow and capillary blood flow. The results suggest that diabetic patients with and without complications have unique peripheral blood flow characteristics.

## Abbreviations

ICG: indocyanine green
BFI: blood flow index
I_max_: maximum intensity
MTT: mean transit time
PWV: pulse wave velocity
DM: diabetes mellitus
PAD: peripheral arterial disease
ABI: Ankle-brachial index
HTN: hypertension
